# Publications Received

**Published:** 1856-05

**Authors:** 


### PUBLICATIONS RECEIVED.

We return our thanks for the following pamphlets:

"Annual (including the medical) Report of the Commissioners of Emigration of the State of New York, for the year ending December 31st, 1855," from which we present some extracts. Under the head of "Hospitals at Ward's Island—their Medical Administration and Statistics for the year," it is stated that "The Hospital Department at Ward's Island has been for several years administered by a Board of Physicians and Surgeons, residing in or near the city, and visiting in rotation, assisted by a number of younger graduates in medicine resident on the island. This system was analogous to that adopted by several other sanitary institutions, and its details will appear more fully in the Annual Reports of the board since 1851. In the judgment of a majority of the Commissioners, there were reasons which rendered this system not well suited to the peculiar situation of these hospitals, though the ability and attention of the visiting physicians were not questioned. In July, 1855, the system was abrogated, and another plan was substituted. The Medical Department proper was placed under the charge of a salaried Physician-in-chief, wholly resident on the Island, with as many salaried assistant physicians, also residing there, as might be required. Dr. H. B. Fay was appointed Physician-in-chief, and Drs. George Ford, and F. Simrock were appointed his assistants. They entered on their duties on 15th August, 1855, and are now in office.

"The Surgical Department was continued under the charge of John Murray Carnochan, M. D., as Surgeon-in-chief, in regard to whose office actual residence was not considered essential. He is required to visit the hospitals at such regular times as prescribed by the by-laws, or when specially needed, and to perform or direct all capital operations. He is assisted by two surgeons, Drs. Darling and Nelken, who reside continually on the island.

"The following summary gives the gross aggregate details of the practice of the year in both departments, and under both systems, during the year. The details as to disease and other matters will be found in the Medical and Surgical Reports appended to this report.

| | |
|---|---|
| There were cared for in the Hospital during the year,... | 11,532 |
| Of whom there died........................................ | 1,043 |
| Were discharged, cured or relieved.................. | 9,713 |
| Remaining at the end of the year..................... | 776 |
| | 11,532 |

"During the preceding years, 1851, 1852, 1853, 1854, there were in the Hospitals 51,314 patients, so that in consequence of the numbers received in the winter of 1855, the whole number in the year exceeds the average of these years one-tenth. Those left at the end of the year are fewer than in any of the preceding years.

"The Refuge Department always contains many aged, infirm or chronically diseased persons, as well as children, not needing regular hospital care, but requiring occasional and often frequent medical attendance. These often pass into hospitals, and many convalescents come from them into the Refuge; so that the whole number, including many counted more than once, of cases receiving medical care, exceeds the number of admissions to the whole establishment.

"The number of cases treated in the Refuge in 1855 was 10,582, of whom 235 died.

"The per centage of deaths in 1855, including both medical and surgical cases in the Hospitals, was, during the first seven and a half months, 9 7-10 per cent. on all treated. For the remaining four and a half months, under the present organization 4 4-5 per cent.

"On the discharges, the rates of mortality was, during the former period, 10 4-5, and for the latter 6⅛.

"It is, however, due to those who administered the department in the former period to add, as observed by the present Physician-in-chief in his report, "that this diminished mortality may have been the result of circumstances other than the change of system;" as the statistics of the city and its institutions show a better state of the general health at one of these periods than at the other. The wards which were filled during the winter, with an average of from 1300 to 1500 patients, had in the latter months of 1855 from 700 to 800, and there was no prevalence of any fatal epidemic. The proportionate decrease was less in the surgical wards, which contained 406 in the beginning of the year, and gradually fell off to 333 at the end.

"The surgical practice has been extensive and successful, including many of the most difficult and important operations in surgery. Some of the details are set forth in the reports appended thereto. During the year there were under treatment 3,517 surgical cases, of which 64 died and 3,120 were discharged cured or relieved—thus showing a proportion of deaths on all cases treated of 1 4-5 per cent., and on all discharged or who died, of two per cent.

"The present condition of the Medical and Surgical Departments is in all respects very satisfactory. The improved insulated hospital wards, erected at different times during the last four years, justify the expectations entertained of their superior comfort and salubrity; whilst the Commissioners are happy to attest to the ability and faithfulness of the Physicians and Surgeons who have charge of these establishments."

Under the head of "Establishment at Castle Garden for the landing place of Emigrant Passengers," we find that "Every facility is provided at the Dépôt, for those whose destination is to the interior, to proceed without unnecessary delay; and without need or pretext for intercourse with the class of persons in the city before alluded to. By this arrangement, much for the benefit of the Emigrant, the Shipper, the Commission and the community at large, has been accomplished. Among these benefits may be mentioned:

"*First.*—To the Emigrants. In a more safe and speedy landing of their person and effects: In the greater safety of their effects after having been put on shore, depredators being limited to fellow-passengers, and but slight opportunity existing for successful pillage by them. In relief from the importunities and deceptions of runners and bookers. In being enabled to continue their journey without delay from the same wharf where they had just landed. In relief from all charges and exactions from landing, "baggage smashing," and porterage; and where they are proceeding to the interior, from cartages. In being enabled to obtain passage tickets at the lowest rates directly from the various transporting companies. In having their baggage accurately weighed; and in being relieved from excessive charges for that which is extra. In obtaining reliable information relative to the various routes of travel throughout the country. In being relieved from the necessity of transporting their baggage to boarding-houses when exigencies require a temporary sojourn in the city of New York. And thus in being enabled to depart for their future homes without having their means impaired, their morals corrupted, and probably their persons diseased.

"*Second.*—To the shipper. In the greater readiness with which passengers are discharged where freight and merchandise do not interrupt the process. In the ship being relieved of its passengers at once, and immediately on arrival. In the consignee being relieved from the supervision of the landing of the passengers.

"*Third.*—To the funds of the Commission. In the increased facility afforded for the discovery of cases liable to special bond. In the opportunity for ascertaining the means of passengers for support. In the reduction of sickness and distress among emigrants. In the diminished proportional number that will become a charge to the Commissioners; and in the means to readily discover paupers and criminals transported hither.

"*Fourth.*—To the Statician. In furnishing reliable data of the fiscal means of emigrants on arrival. In developing the points of individual destination; thus exhibiting the number of persons who, at the time of arrival, are destined for each State, and the money means with which they are provided.

"*Fifth.*—To the community in general. In the diminution of human suffering. In the reduction of calls on the benevolent throughout the country; and in the dispersion of a band of outlaws attracted to this port by plunder, from all parts of the earth.

"All these results are stated not as merely probable, but as results actually and permanently obtained, the evidence of which is constantly passing under the observation of the Commissioners and of the merchants and others immediately connected with the commerce and navigation of the port. The tables accompanying the report will show in part the working of this system."

"An address to the graduating class of the fifth session of the University of Nashville, by John P. Ford, M. D., which we regret that we have not had time to read, as from a slight glance, it seems to be a creditable production.

"Treatment of displacements of the uterus with the abdominal spring pessary, by J. F. McGaston, M. D.," which we shall probably publish, and therefore defer any comment upon it for the present.

"A paper on the effects of lead on the heart, by Jno. W. Corson, M. D., New York." It is doubtless a valuable paper, and we give a short extract containing some practical observations worthy of attention. He remarks: "We have before alluded, in passing, to many known or unsuspected methods of exposure to lead. In illustration, we may simply add the following list from the great work of Tanquerel des Planches: "Of 101 subjects of lead paralysis, there were manufacturers of white lead, 31; do. of minium, 6; painters of buildings, 22; do. of carriages, 4; do. ornamental, 5; grinders of colors, 6; manufacturers of German cards, 1; potters, 5; refiners, 3; plumbers, 3; type founders, 4; printers, 3; lapidaries, 3; cutters of crystals, 1; manufacturers of acetate of lead, 2; do. sulphate of lead, 1; do. chromate of lead, 1."

"In experiments detailed in the *Edinburgh Medical and Surgical Journal*, Mr. Blake found that a drachm of the acetate of lead taken into the stomach of a dog, suddenly arrested the heart's action, and that the small quantity of three grains injected into the jugular vein, diminished the force of the heart.

"Foidère, in the post-mortem examination of a patient with lead disease, describes the heart as presenting a shrunken or 'withered' appearance.

"Again, setting aside the dispute about 'natural lead,' the chemical researches of Orfila, Tiedeman, Gmelin, Devergie, and Guibort, have detected in subjects who have died of lead disease, an unusual amount of the poisonous metal in the brain muscles, thoracic and abnominal viscera, and especially in the blood.

"Now, as every-day practice proves, even when medicinally applied to the skin, lead is a sedative. Let its poison course along the lining membrane of the arteries and veins, and thus mingling with the blood, its vital stimulant, bathe constantly the central organ of circulation, and we can easily see why the heart should especially feel its paralyzing influence.

"*Treatment.*—The chief remedies to counteract the depressing effects of lead may be divided into two classes. The first may be termed *disinfectants*, such as the iodide of potassum, and the various preparations of sulphur; and these act by eliminating the poison from the system, and thus remove the causes.

"The second class—if we may coin a word easily understood —may be designated *Anti-paralytics*, such as strychnia and electricity. These restore tone to the injured organs, and thus powerfully relieve effects."

Also "A Brief History of the Origin, Progress and Extension of the Epidemic Yellow Fever in Memphis, Tenn., in 1855, &c., &c.," a pamphlet of some 40 pages of interesting matter, deserving a more extended notice than we can give it at present, which we regret the more, as our estimate of the author, both indivi [illegible]ually and professionally, is decidedly favorable. It is from the pen of L. Shanks, M. D., Professor of Obstetrics and Diseases of Females, in the Memphis Medical College.

And "An Essay on Intermittent and Bilious Remittent Fevers, with their Pathological Relation to Ozone," by E. S. Gaillard, M. D., which we will notice more particularly, when we have had an opportunity of reading it.

The report of the Board of Managers of the Eastern Lunatic Asylum, (at Lexington, Kentucky,) for the years 1854-'5.

And lastly, a pamphlet of some 24 pages containing some interesting and valuable information in reference to the "Medical Properties of Bailey's Spring, Lauderdale County, Alabama, and Cooper's Well, Hinds County, Mississippi, by S.

C. Farrar, M. D., of Jackson, Mississippi." From the testimony presented, these are doubtless valuable waters. We conclude with an extract from these pages, containing some judicious and practical observations in relations to a point, the non-appreciation of which, upon the part of our patients, and in many instances, upon the part of the physician, presents the prominent obstacle in the way of treating chronic diseases generally, and more particularly those of the digestive organs, he remarks: "The reader will pardon us if we suggest the importance of temperance in eating and drinking, to those who visit the spring for the benefit of the water. We are convinced that many persons use mineral waters too freely, from the supposition, that the larger the quantity, the more certain and speedy will be the cure. This is a great mistake. We think from six to twelve glasses of this water are sufficient for one day, but we have heard of persons going there who consumed from twenty to thirty, as if the human stomach possessed the capacity of distention given to the camels. Others err in diet. No sooner is the appetite improved, than they lay aside all restraint, and eat as if a wager depended on the amount of food consumed. Some people have a very vague and undefined idea of temperance in eating. [illegible]s an illustration of the fact, we will advert to an anecdote that occurred many years ago, between that eminent but eccentric European physician, Mr. Abernethy, and one of his patients: A gentleman called on Mr. Abernethy to get his medical advice. He entered into a long history of his disease, and related all its particulars. When he concluded, 'Perhaps,' said the sagacious physician, 'you eat too much.' The man avowed that he did not. 'But,' replied Mr. Abernethy, 'what do you take for breakfast, and what for dinner, and what for supper?' The patient went on to recount a long list of dishes, undoubtedly showing that his unpleasant sensations resulted from intemperance in eating. 'Well,' said Mr. Abernethy, 'go home and eat less.' And to many who visit watering places for health, we would beg leave to say, eat less. A hint to the wise is sufficient."

We have also to acknowledge within a day or two, the reception of the following medical works, from the publishers, Messrs. Blanchard & Lea, Philadelphia, through Messrs. J. J.

Richards & Co., booksellers of this City, who are receiving a supply of new and valuable medical works, and from whom we are satisfied they can be obtained on favorable terms. We shall notice these works more particularly in the next number of the Journal: "Flint on the Respiratory Organs," "Brown on Surgical Diseases of Women," "Neligan's Atlas of Diseases of the Skin," "Neil & Smith's Compend. of Medicine," Lehman's Chemical Physiology.

